# Composite tumor with pheochromocytoma and immature neuroblastoma: report of two cases with cytogenetic analysis and discussion of current terminology

**DOI:** 10.1007/s00428-017-2225-9

**Published:** 2017-09-01

**Authors:** Lily Tran, Carrie Fitzpatrick, Susan L. Cohn, Peter Pytel

**Affiliations:** 1 Department of Pathology, University of Chicago, 5841 S. Maryland Ave, Chicago, IL USA; 2 Department of Pediatrics, University of Chicago, 5841 S. Maryland Ave, Chicago, IL USA

## Introduction

Pheochromocytoma, paraganglioma, and neuroblastoma are all derived from sympathetic nervous tissues and can exhibit a spectrum of morphology. The literature includes reports of tumors with morphologic features of both pheochromocytoma and neuroblastoma within the same tumor. The term “composite pheochromocytoma” has been used to describe these tumors as well as pheochromocytomas with other neural crest derivatives, such as malignant peripheral nerve sheath tumor and neuroendocrine carcinoma. “Composite pheochromocytoma” is rare, and there is a paucity of information in the literature regarding its biological behavior, clinical outcome, and molecular profile. The most commonly reported cases include components of pheochromocytoma and ganglioneuroma [1]. However, there are only few reports of tumors with components of pheochromocytoma and stroma-poor neuroblastoma (Table 1) [2–7]. We report two distinct cases of composite tumors of the adrenal gland consisting of pheochromocytoma and neuroblastoma.

**Table 1:** Summary of previously reported cases of composite pheochromocytoma with immature neuroblastoma component including the two cases reported in this study

Case number	Reference	Age (years)/sex	Associated syndromes	Location	Metastasis	Treatment	N-MYC amplification	Follow-up
1	Wahl 1943 [2]	4/M	None	Mediastinum	Yes	Radiation	N/A	Died (8-month follow-up)
2	Franquemont 1994 [3]	49/M	None	Adrenal-left	No	Surgery	N/A	No evidence of disease after 1 year
3	Franquemont 1994 [3]	38/M	None	Adrenal-right	Yes	Surgery and chemoradiation	N/A	Died 3 months after diagnosis
4	Candanedo-Gonzalez 2001 [4]	56/F	None	Adrenal-right	No	Surgery	N/A	Asymptomatic at 24 months post-op
5	Tatekawa 2006 [5]	5/M	None	Adrenal-left	No	Surgery	N/A	Unknown
6	Comstock 2009 [6]	17/M	NF1	Adrenal-laterality unspecified	No	Unknown	Non-amplified	Alive (follow-up date unknown)
7	Steen 2014 [7]	46/M	None	Adrenal-left	No	Surgery	N/A	Asymptomatic and normotensive at 6 months post-op
8	Current study	57/M	NF1	Adrenal-left	No	Surgery	Non-amplified	Asymptomatic and normotensive (6 months post-op)
9	Current study	5/M	None	Adrenal-left	Yes	Surgery, chemoradiation, SCT	Amplified	Died 2 years after diagnosis

*NF 1* neurofibromatosis type 1, *SCT* stem cell transplant

## Methods

Purified DNA was analyzed using the OncoScan™ Affymetrix microarray platform following the manufacturer’s instructions (Affymetrix Inc., Santa Clara, CA). The copy number and genotype data were analyzed using the GeneChip workstation with Affymetrix Molecular Diagnostics Software. OSCHP files were analyzed using Nexus Express Software, Biodiscovery v3.1. Genome build hg19 (Feb 2009) was used for probe locations and data interpretation.

## Case report

### Case 1

**Fig. 1:**
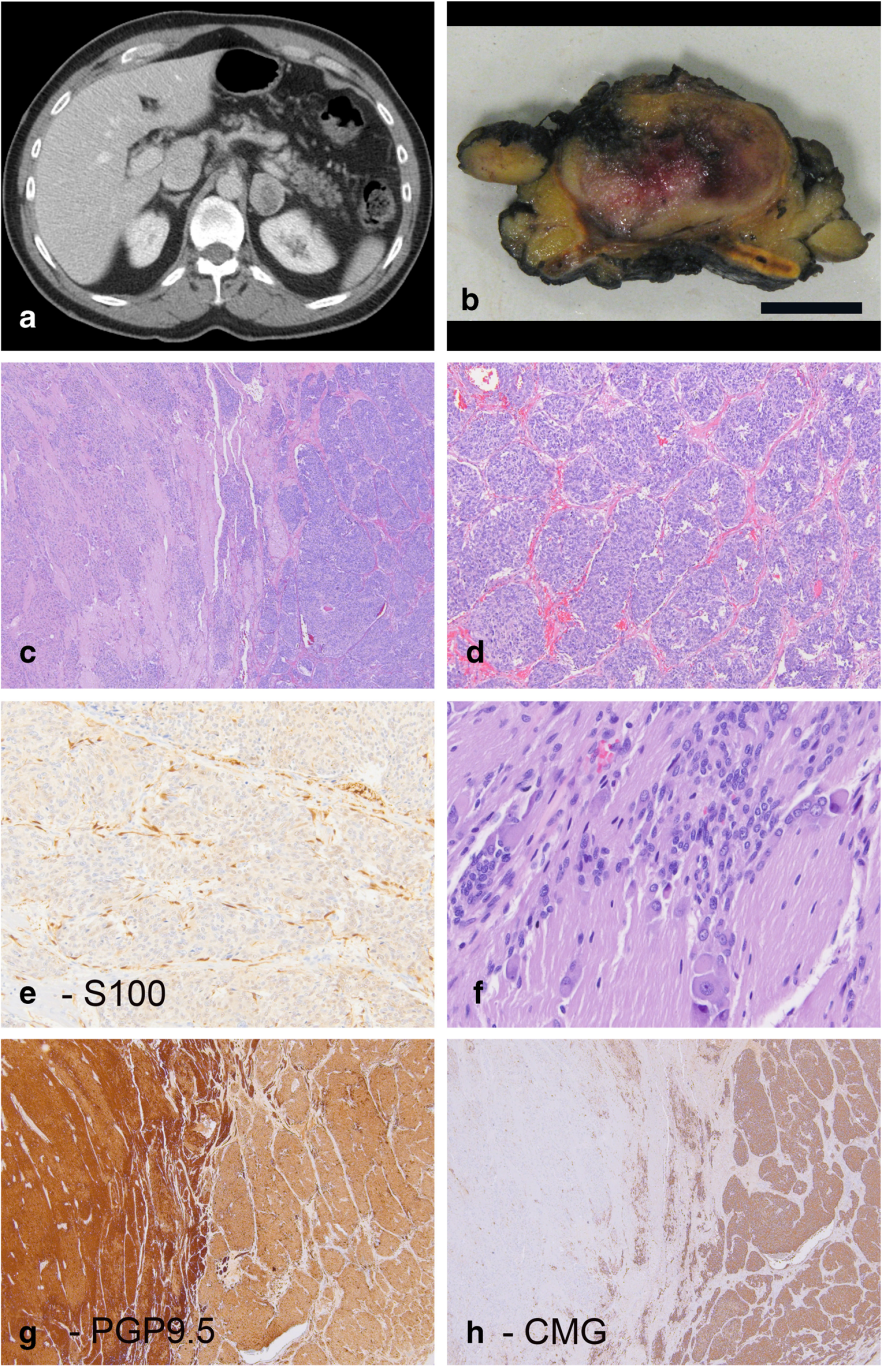
Patient 1 was a 57-year-old male who was incidentally discovered to have a left adrenal mass lesion on a CT scan performed for hematuria (**a**). On cross section, the tumor consisted of a demarcated lesion arising within the adrenal gland (**b**) (size bar 1 cm). H&E images show a biphasic lesion (**c**) with nested areas of pheochromocytoma (**d**) that includes S100 positive sustentacular cells (**e**). Other areas show neuropil-type stroma with immature small neuroblastic cells and rare ganglion cells (**f**). PGP9.5 and chromogranin corresponding to the same area seen in panel C show distinct labeling in the two components. The neuroblastoma component is more strongly positive for PGP9.5 (**g**) while chromogranin (CMG) expression is largely restricted to the pheochromocytoma component of the tumor (**h**)

A 57-year-old male, who was initially diagnosed with neurofibromatosis type 1 (NF1) after exhibiting facial neurofibromas at age 20, presented with hematochezia. Workup, including CT and MRI, revealed an incidental 3.6 cm enhancing mass in the left adrenal gland (Fig. 1). A 24-h urine catecholamine collection was obtained which showed an elevated dopamine level of 1015 μg/24 h (reference range 52–480 μg/24 h), and blood work revealed a plasma normetanephrine level of 2.53 nmol/L (reference range 0–0.89 nmol/L). The patient denied headaches, elevated blood pressure, diaphoresis, shortness of breath, and tachycardia.

He underwent a left adrenalectomy. Grossly, the tumor formed a white to yellow, beige circumscribed mass with patchy hemorrhage. No distinct areas or nodules were appreciated on the gross exam (Fig. 1b). Histologic sections showed an intimate admixture of two distinct components (Fig. 1c–h). The first component exhibited features of a pheochromocytoma with nests of epithelioid to spindled cells. The periphery of the nests contained S100 and SOX10 positive sustentacular cells. The second component contained fine fibrillary neuropil-type stroma and cells that varied from small neuroblastic to focal ganglion cells. Both components stained for PGP9.5, synaptophysin, and chromogranin but with variable intensities: the neuroblastic component was strongly and diffusely positive for PGP9.5 and only exhibited weak labeling for chromogranin, while the reverse was true for the pheochromocytoma component. Overall, the pheochromocytoma component constituted 60% of the tumor while the neuroblastic component constituted 40%.

Tissue from each component was dissected from unstained slides matched to a serial hematoxylin and eosin (H&E) stained section. Whole genome SNP arrays were performed to assess genome wide copy number aberrations (CNAs) and copy neutral regions of loss of heterozygosity (LOH). Identical gains and losses of the following regions were identified in both tumor components (supplement 1): del(1p), del(4p), dup(4q), del(5q), del(6q), del(9p), del(17p), and trisomy 18.

Six months post-operatively, the patient showed no evidence of recurrence.

### Case 2

A 5-year-old previously healthy male presented with abdominal pain. An X-ray demonstrated calcifications in the left hemi-abdomen extending from the left renal fossa laterally. This was associated with permeative lytic lesions involving the proximal femora and right ischium. He underwent a biopsy of an adrenal mass as well as bilateral bone marrow biopsies. The bone marrow biopsies were positive for involvement by neuroblastoma, and the biopsy of the adrenal mass showed features of a neuroblastic tumor with focal areas of cells in a nested arrangement. At central review, the tumor was classified as a “composite pheochromocytoma” in accordance with the current terminology used in the World Health Organization tumor classification guidelines [8]. Biologic studies were positive for *MYCN* amplification. After high-risk neuroblastoma induction therapy, a tumor resection was performed. The patient was then treated with high-risk therapy and a stem cell transplant followed by radiation and isotretinoin. Two years after the initial therapy, the patient developed treatment-refractory metastatic disease and died.

**Fig. 2:**
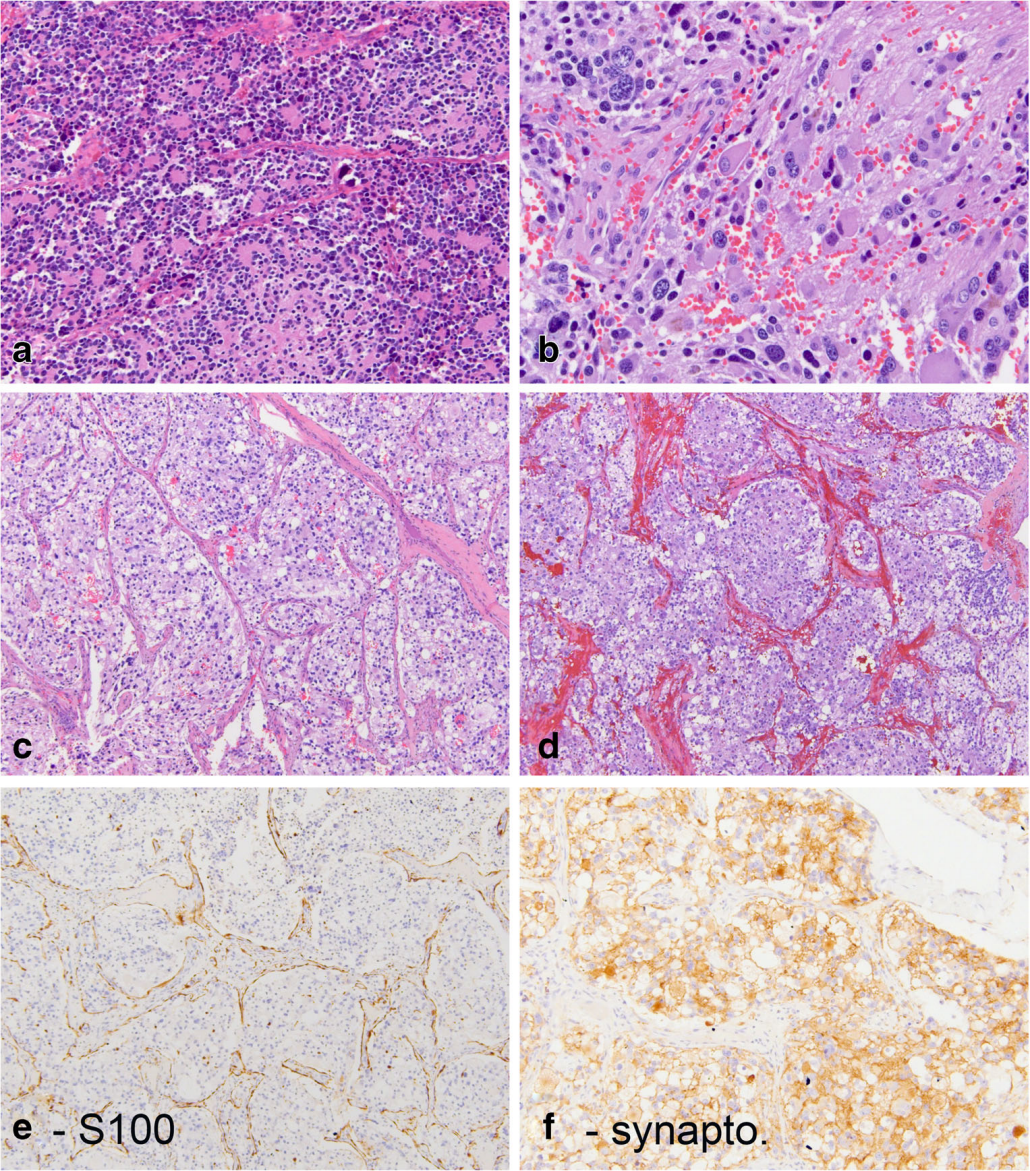
Patient 2 was a 5-year-old boy who presented with an adrenal lesion that contained two intimately admixed histomorphologic components. Some areas (**a**, **b**) show typical neuroblastoma with focal rosette-like arrangement of immature cells and focal neuropil stroma. Other areas have a distinctly nested arrangement of cells with more abundant cytoplasm (**c**, **d**) and distinct peripheral staining for S100 in a pattern seen with sustentacular cells (**e**). Synaptophysin is positive in these latter areas (**f**)

Grossly, the tumor showed a homogeneous surface without distinct nodules. Histologic sections of the resected adrenal tumor revealed similar features as those observed in the pre-treatment biopsy (Fig. 2). Some areas exhibited features of neuroblastoma, including immature neuropil-type stroma and immature neuroblastic cells with isolated cells showing focal ganglion cell differentiation. Other areas contained epithelioid cells with relatively abundant cytoplasm in a nested arrangement. In these latter areas, S100 staining highlighted the presence of delicate spindled sustentacular cells around the tumor nests.

In the post-treatment resection specimen of the adrenal gland, approximately 10% of the specimen was composed of necrotic tumor. Both the neuroblastoma and pheochromocytoma components were present in multiple tissue blocks. The neuroblastic component comprised 70% of the tumor, and the pheochromocytoma component comprised the remaining 20%. Whole genome SNP arrays were attempted in this case also but failed to yield diagnostic results.

## Discussion

Tumors with morphologic features of pheochromocytoma and stroma-poor neuroblastoma in the same lesion are rare and are commonly referred to as “composite pheochromocytoma.” Table 1 summarizes the clinical features of published cases [2–7]. We report two additional cases for which we provide further molecular characterization. Morphologically, both cases share a biphasic appearance with areas of immature stroma-poor neuroblastoma and areas of pheochromocytoma comprised of cells in a nested arrangement outlined by sustentacular cells.

In the first case, SNP microarray results suggest that the two components are clonal since they share the same pattern of chromosomal gains and losses. Furthermore, these gains and losses are largely in concordance with prior published reports for pheochromocytoma [9–11]. The SNP microarray primarily captures large chromosomal changes and is unable to detect mutations, genomic rearrangements, or epigenetic changes. The morphologic difference between the two tumor components may therefore still be associated with and reflective of molecular changes that are not detected by this platform.

Despite the unusual morphologic findings, this first case of an adult with NF1 and an incidentally discovered indolent lesion bears all the clinical features of a pheochromocytoma. This is in contrast to the second case of a young child with a *MYCN*-amplified tumor that was associated with diffuse bone marrow involvement at presentation. His clinical course was characteristic of high-risk neuroblastoma: he initially responded to high-risk neuroblastoma treatment but subsequently developed relapsed disease that was refractory to treatment, eventually leading to the patient’s demise.

A review of the previously published cases of composite tumors with neuroblastic and pheochromocytoma components shows that these cases largely fall into two groups that recapitulate the characteristics of the two reported cases. As shown in Table 1, cases 2, 4, 6, and 7 are adult patients or patients with NF1, similar to the first case we are reporting. In contrast, case 1 in Table 1, is a 4-year-old with rapidly fatal disease, comparable to our second case. No follow-up information is provided for case 5. Case 3 represents an outlier, since this 38-year-old patient died with widely metastatic disease within 3 months of diagnosis. With the exception of the case reported by Comstock et al. which demonstrated no N-MYC gene amplification in the tumor of their NF1 patient [6], other studies did not investigate this important prognostic factor [2–5, 7].

The term “composite neuroblastoma” or “composite tumor” has been applied in the context of neuroblastic tumors to describe those rare cases that appear comprised of morphologically distinct clones of neuroblastoma [12–14] while the term “composite pheochromocytoma” has been adopted as a descriptor for tumors exhibiting features of neuroblastoma and pheochromocytoma as illustrated by the two cases reported here. Nevertheless, the term “composite pheochromocytoma” has largely been used in a non-discriminatory way, regardless of the underlying biology and clinical setting and may, therefore, be misleading for treating physicians. A terminology based on the biology and clinical setting of the tumor is likely to be less ambiguous. Our first case may best be described as “pheochromocytoma with divergent components of neuroblastoma differentiation” while the second case would more accurately be described as “neuroblastoma with divergent components of pheochromocytoma differentiation.” Divergent differentiation is observed in various tumors including dedifferentiated liposarcoma and malignant peripheral nerve sheath tumors. One can speculate on the implications of this phenomenon for cell fate, but ultimately this question is not resolved [15]. However, the distinction between neuroblastoma and pheochromocytoma is clinically important because patients with high-risk neuroblastoma require multimodal therapy while those with pheochromocytomas are often cured with surgical resection alone.

## Electronic supplementary material

Supplemental 1 Summary of the (identical) copy number aberrations detected in the pheochromocytoma and neuroblastic components of Case 1. To exclude germline CNVs, gains and losses <400 Kb were filtered by software. Genomic coordinates correspond to genome build 19. (DOCX 72 kb)
