# Supplementary material for: Composite tumor with pheochromocytoma and immature neuroblastoma: report of two cases with cytogenetic analysis and discussion of current terminology
Source: Virchows Arch. 2017 Sep 1;471(4):553–7. doi: 10.1007/s00428-017-2225-9 (PMC5614909; doi:10.1007/s00428-017-2225-9)
Supplement: Supplementary file 1 — Summary of the (identical) copy number aberrations detected in the pheochromocytoma and neuroblastic components of Case 1. To exclude germline CNVs, gains and losses <400 Kb were filtered by software. Genomic coordinates correspond to genome build 19. (DOCX 72 kb) [file 428_2017_2225_MOESM1_ESM.docx]

| **Chromosome Region (hg19)** | **Event** | **Length (bp)** | **Cytoband** | **Probe Median (log_2_ fold change)** | **Probe number** | **Cosmic entries** |
| --- | --- | --- | --- | --- | --- | --- |
| chr1:754,192-158,265,687 | CN Loss | 157511496 | p36.33 - q23.1 | -0.487550348 | 10803 | 1,275 |
| chr4:136,191,040-190,915,650 | High Copy Gain | 54724611 | q28.3 - q35.2 | 0.615590781 | 4080 | 171 |
| chr4:69,404-49,092,454 | CN Loss | 49023051 | p16.3 - p11 | -0.498986438 | 3344 | 201 |
| chr5:158,731,548-180,698,312 | CN Loss | 21966765 | q33.3 - q35.3 | -0.473388374 | 1597 | 142 |
| chr5:82,505,153-158,462,918 | CN Loss | 75957766 | q14.2 - q33.3 | -0.50056994 | 5333 | 386 |
| chr6:111,726,146-170,913,051 | CN Loss | 59186906 | q21 - q27 | -0.516303986 | 4558 | 250 |
| chr9:204,738-44,893,094 | CN Loss | 44688357 | p24.3 - p11.2 | -0.515149951 | 3229 | 202 |
| chr17:400,959-42,415,772 | CN Loss | 42014814 | p13.3 - q21.31 | -0.469314456 | 3375 | 632 |
| chr18:12,842-78,007,784 | CN Gain | 77994943 | p11.32 - q23 | 0.338173687 | 5595 | 251 |
|  |  |  |  |  |  |  |
